# Correction: Visible-light acridinium-based organophotoredox catalysis in late-stage synthetic applications

**DOI:** 10.1039/d4ra90011a

**Published:** 2024-02-13

**Authors:** Praveen P. Singh, Jaya Singh, Vishal Srivastava

**Affiliations:** a Department of Chemistry, United College of Engineering & Research Naini Prayagraj 211010 India ppsingh23@gmail.com; b Department of Chemistry, LRPG College Sahibabad Gaziabad Uttar Pradesh India; c Department of Chemistry, CMP Degree College, University of Allahabad Prayagraj 211002 Uttar Pradesh India vishalgreenchem@gmail.com

## Abstract

Correction for ‘Visible-light acridinium-based organophotoredox catalysis in late-stage synthetic applications’ by Praveen P. Singh *et al.*, *RSC Adv.*, 2023, **13**, 10958–10986, https://doi.org/10.1039/D3RA01364B

*RSC Advances* is issuing this correction to notify readers that there are portions of text overlap with a number of different sources, and the text should have been rewritten to avoid the overlapping text. In addition, the authors regret that part of Section 5.23 was written incorrectly and should have been referenced to make it clear that this mechanism was described by Nicewicz and co-workers.

It should be shown as below:

The plausible mechanism as proposed by Nicewicz and co-workers is depicted in Scheme 39. According to this mechanism, if “ketone first” reduction is operative, a ketyl radical **72A** is formed, which can undergo a radical 5-*exo-trig* cyclization with the corresponding olefin to provide a carbon-centered radical **72C**. Then, terminal HAT can occur to give the corresponding cyclized product **73**. Alternatively, when “olefin first” reduction occurs, they proposed that the olefin radical anion **72B** formed, can undergo a two-electron attack at the carbonyl to generate **72C**. Subsequent HAT from either DIPEA or 1,4-CHD can trap out the corresponding cycloadduct **73**.^67^

The Royal Society of Chemistry apologises for these errors and any consequent inconvenience to authors and readers.

## Supplementary Material

